# GPR109A activation and aging liver

**DOI:** 10.18632/aging.102343

**Published:** 2019-10-07

**Authors:** Ravirajsinh N. Jadeja, Pamela M. Martin

**Affiliations:** 1 Department of Biochemistry and Molecular Biology, Augusta University, Augusta, GA 30912, USA; 2 James and Jean Culver Vision Discovery Institute, Augusta, GA 30912, USA; 3 Georgia Cancer Center, Augusta University, Augusta, GA 30912, USA; 4 Department of Ophthalmology, Medical College of Georgia at Augusta University, Augusta, GA 30912, USA

**Keywords:** aging, steatosis, GPR109A, lipid metabolism

GPR109A [hydroxycarboxylic acid receptor 2 (HCAR2), HM74A or PUMA-G] is expressed in various cells and tissue types including adipocytes, keratinocytes, immune cells, and epithelial cells (colon and retina) [1,2]. On activation, it regulates metabolic and immune signaling in various cell types. Despite the essential role of GPR109A receptor as a metabolic sensor, its expression and function in the liver was not fully understood until recently. In fact, several earlier studies reported that the receptor was not expressed at all in the liver [1] or, that is expressed in the tissue but only minimally [3]. In keeping with the long-standing interest of our research group in understanding GPR109A signaling, in one of our recent studies, we examined GPR109A expression in normal human and mouse livers and in various liver cell types [4]. We also evaluated the impact of GPR109A deletion on age-associated hepatic steatosis. We found GPR109A to be expressed in human and mouse liver tissues, with Kupffer cells displaying the highest level of expression. Importantly, we additionally demonstrated hepatic GPR109A expression to decline with age in mice a phenomenon that we speculated might contribute to increase fat accumulation in aging. This we confirmed using GPR109A^-/-^ mice, and indeed we saw that loss of expression of GPR109A was correlated with the enhanced accumulation of visceral and hepatic fat in aged mice [4]. Our findings collectively provided extensive evidence for GPR109A expression in hepatocytes/liver and its importance in controlling *de novo* lipogenesis in liver and adipose tissue of aging mice.

A few years after the first report on niacin’s lipid lowering efficacy, GPR109A was identified as its receptor. Since then the use of niacin to treat dyslipidemia has been well documented. While, some of these studies showed involvement of GPR109A, others reported GPR109A independent effects [5]. Recently, a study by Ye et al., 2019 provided further evidence that GPR109A is expressed in liver and hepatocytes [6]. They additionally showed that niacin-induced GPR109A signaling fine-tunes lipid metabolism through various mechanisms, including the inhibition of hepatocyte lipogenesis and fatty acid intake, and the promotion of brown adipose tissue thermogenesis. Although, initial studies concluded that niacin’s positive effects on lipid metabolism are independent of GPR109A, studies by Ye and colleagues [6] in combination with our study [4], provide conclusive evidence of the role of GPR109A as nutrient sensor in liver and the related protective effects of its activation in reducing hepatocyte fat accumulation. As the debate continues over the favorable effects of niacin-GPR109A on the development and progression of dyslipidemia [7], it will be interesting to perform further studies on assessing the role of niacin-induced GRP109A activation in regulating aging-associated hepatic statosis. Additionally, monomethylfumarate the principal bioactive component of an FDA approved therapy (Tecfidera, Biogen IDEC) is a GPR109A agonist [8] that can also potentially be repurposed to treat fatty liver diseases.

Several years following the discovery of GPR109A, β-hydroxybutyrate (BHB), a ketone body produced by the liver, was identified as an endogenous ligand. Recent studies have shown that BHB, a natural ligand for GPR109A is an important signaling molecule that regulates various metabolic pathways. Various dietary approaches such as the use of a ketogenic diet, intermittent fasting and calorie restriction have been shown to increase circulating levels of BHB. Hence, the therapeutic approach of activating GPR109A receptor has a highly translational value as some of the GPR109A agonist drugs are already approved for human use, and use of ketogenic diet, intermittent fasting and calorie restriction has shown great benefits in improving other age-associated abnormalities. Therefore, these therapeutic approaches will not only have application in ameliorating hepatic steatosis in aged patients, but may also be beneficial for the treatment of other co-morbidities (e.g. Age-related macular degeneration, Alzheimer’s disease, Parkinson’s disease etc.). Thus, future studies to determine efficacy of these dietary manipulations in regulating aging-associated hepatic steatosis and related liver conditions are sorely needed.
